# Extreme dislocation of the cervical spine—case report

**DOI:** 10.1093/jscr/rjae039

**Published:** 2024-02-06

**Authors:** Slavisa Zagorac, Milos Vasic, Uros Novakovic, Milos Mladenovic, Ivan Tulic, Valerija Teodosic, Dragana Vracevic

**Affiliations:** Faculty of Medicine, University of Belgrade, dr Subotica starijeg 8, Belgrade 11000, Serbia; Clinic for Orthopedic Surgery and Traumatology, University Clinical Center of Serbia, Pasterova 2, Belgrade 11000, Serbia; Clinic for Orthopedic Surgery and Traumatology, University Clinical Center of Serbia, Pasterova 2, Belgrade 11000, Serbia; Clinic for Orthopedic Surgery and Traumatology, University Clinical Center of Serbia, Pasterova 2, Belgrade 11000, Serbia; Clinic for Orthopedic Surgery and Traumatology, University Clinical Center of Serbia, Pasterova 2, Belgrade 11000, Serbia; Clinic for Orthopedic Surgery and Traumatology, University Clinical Center of Serbia, Pasterova 2, Belgrade 11000, Serbia; Clinic for Orthopedic Surgery and Traumatology, University Clinical Center of Serbia, Pasterova 2, Belgrade 11000, Serbia; Clinic for Orthopedic Surgery and Traumatology, University Clinical Center of Serbia, Pasterova 2, Belgrade 11000, Serbia; Department of Anesthesiology, Reanimation and Intensive Care, Clinic for Orthopedic Surgery and Traumatology, University Clinical Center of Serbia, Pasterova 2, Belgrade 11000, Serbia

**Keywords:** traumatic C6/C7 dislocation, transection of the spinal cord, timing of the surgical procedure, surgical approach

## Abstract

We present the case of rare extreme dislocation of subaxial cervical spine, which was challenging regarding type and time of surgery. A 22-year-old patient was injured in a traffic accident, from very beginning with signs of spinal shock. Severe traumatic C6/C7 dislocation with resulting transection of the spinal cord was diagnosed with MDCT imaging. The main dilemmas regarding the surgical treatment of this injury referred to the timing of surgery and the choice of surgical approach. We decided to perform posterior surgery at first stage. Postoperative her condition get worsening and on the 16th postoperative day came to the fatal outcome. Despite all the available protocols, in our case, the decision had to be made on the basis of individual multidisciplinary assessment, bearing in mind the mechanism of the injury and the clinical presentation of the injured patient.

## Introduction

Due to its particular anatomy (mobility) and function, the cervical spine is subject to a variety of injuries, from muscle strains to complete dislocation, while a significant number of injuries are associated with spinal cord injury and resulting neurological deficit. The severity of the injury and treatment depends on injury and patient related factors [1]. Cervical spine injuries can be roughly categorized as axial spine injuries (occiput, C1 and C2), and the children are prone to suffer dislocation at this level [2], and subaxial spine injuries (C3–C7), where traumatic dislocations occur more common [3, 4]. Recovery from neurological deficit is uncertain, even after the administration of appropriate medication and surgical treatment [5]. The most common mechanism of traumatic cervical vertebrae dislocation is hyperextension or hyperflexion with distraction, which occurs most often in traffic accidents [6].

We present a case of extreme traumatic dislocation at the C6/C7 level with complete transection of the spinal cord, in a young girl who was riding in a car as a front seat passenger.

## Case presentation

A 22-year-old female patient was injured in a traffic accident while riding in the front passenger seat of a car. She was immediately intubated, transport cervical immobilization was applied, supportive therapy was administered, and the patient was transported to the University Clinical Center of Serbia Emergency Center in Belgrade, which is the main trauma center in Serbia.

**Figure 1:**
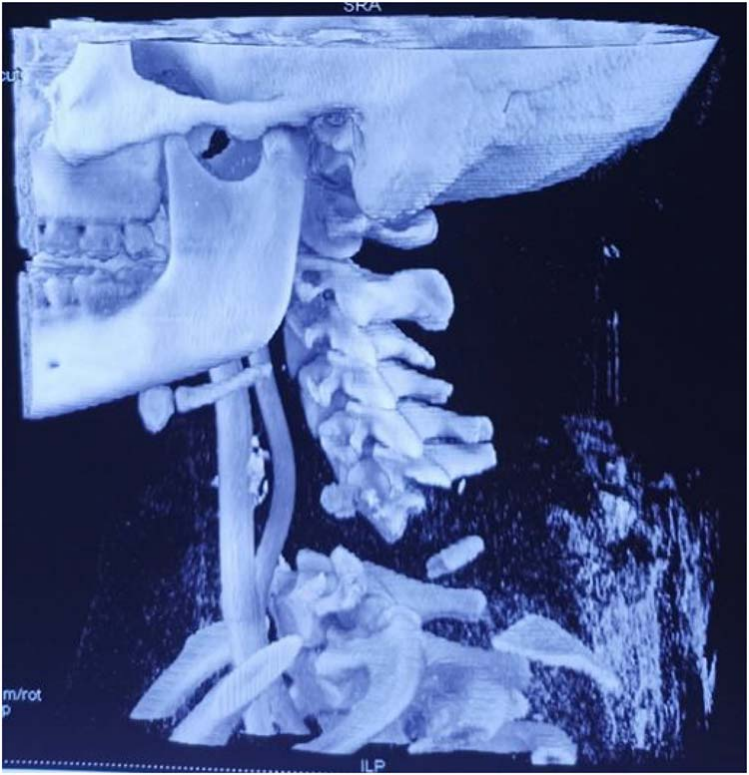
Computed tomography finding (3D reconstruction): C6/C7 dislocation.

**Figure 2:**
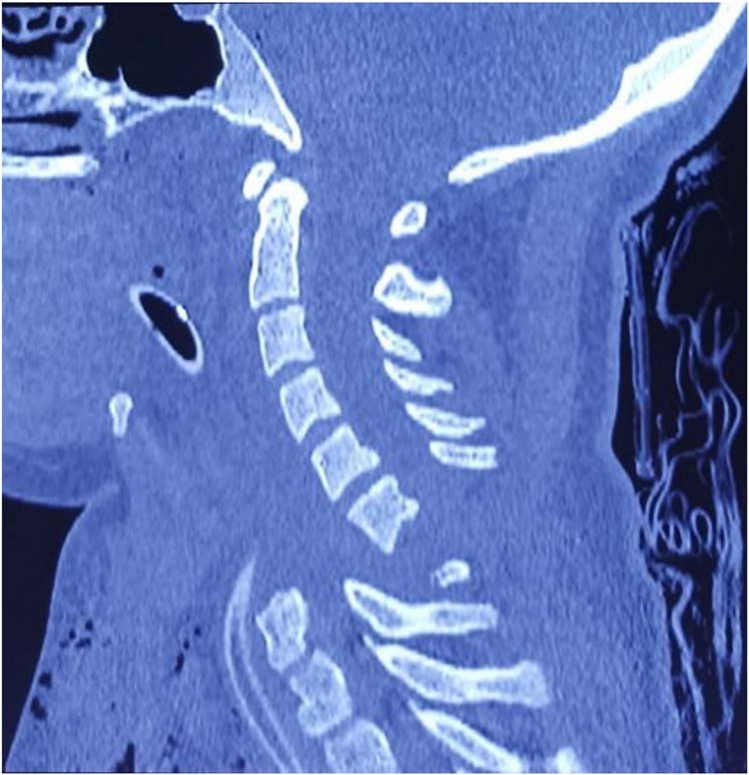


Computed tomography revealed complete traumatic dislocation of C6/C7 with consequent transection of the spinal cord (Figs 1 and 2).

Upon hospital admission, the patient was in life-threatening condition, hemodynamically unstable, on vasopressor support, continuous analgesia, on antibiotic therapy and on high doses of corticosteroids, with a large hematoma and skin abrasions in the region of the upper third of the sternum and neck.

This injury required urgent surgical treatment – reduction and stabilization. However, upon admission, there was an extremely high risk of intraoperative and perioperative complications.

**Figure 3:**
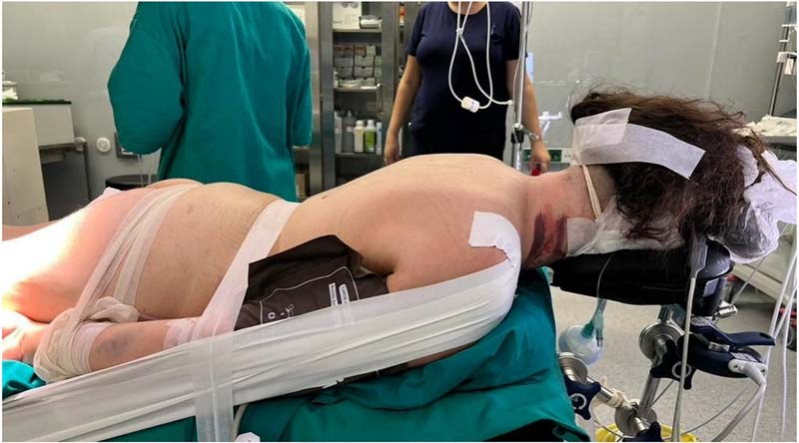
Position of the patient during surgery – posterior approach.

**Figure 4:**
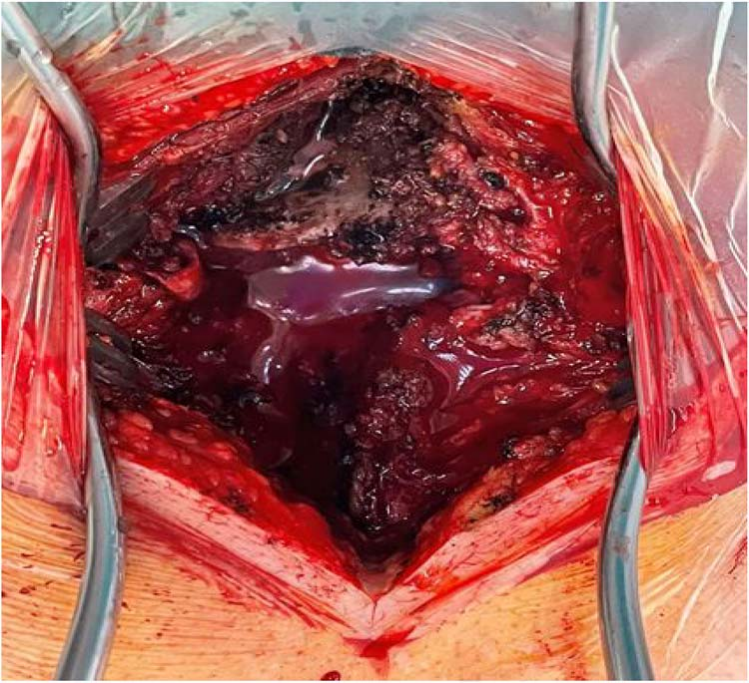
Intraoperative finding: C6/C7 dislocation.

**Figure 5:**
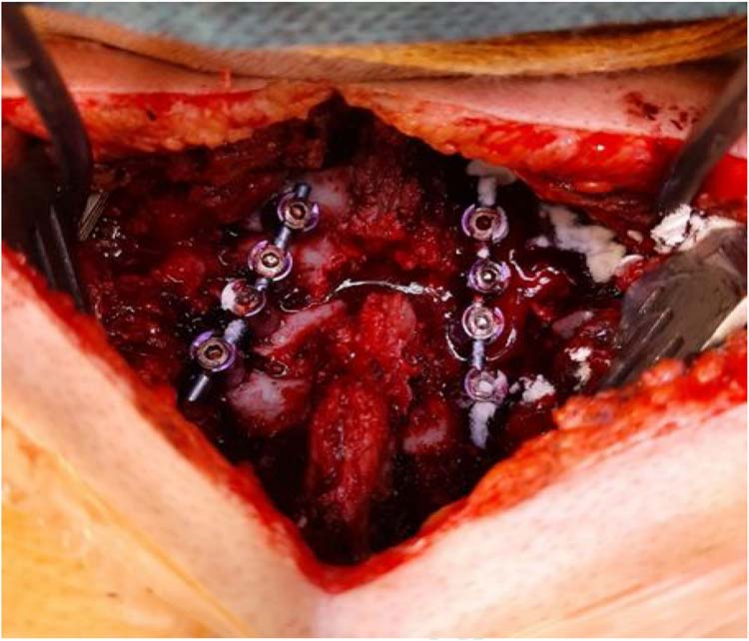
Intraoperative finding after repositioning and stabilization: screws placed into the C5-C7 massae laterales and the T1 pedicles; fixation performed with titanium rods.

**Figure 6:**
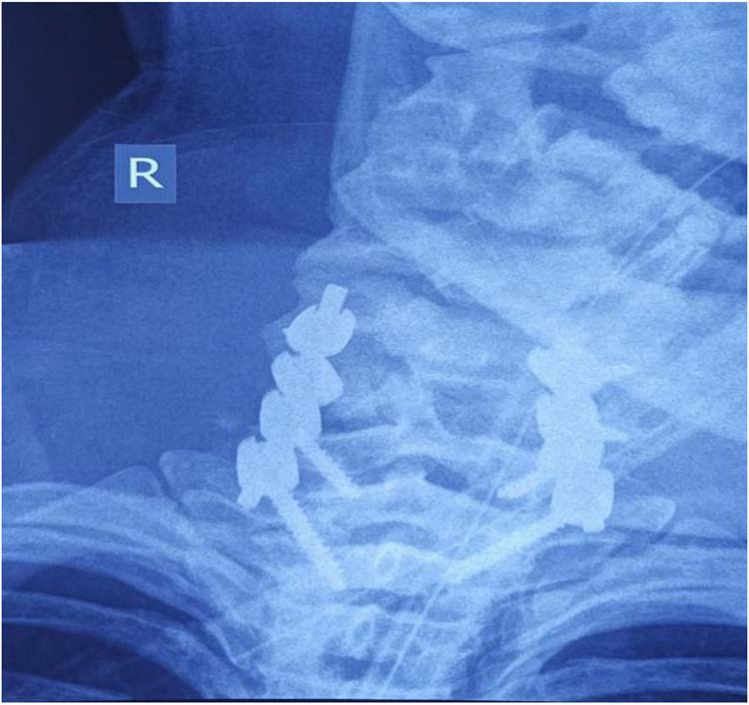
Cervical spine X-ray postoperatively – AP view: properly positioned osteosynthesis material.

**Figure 7:**
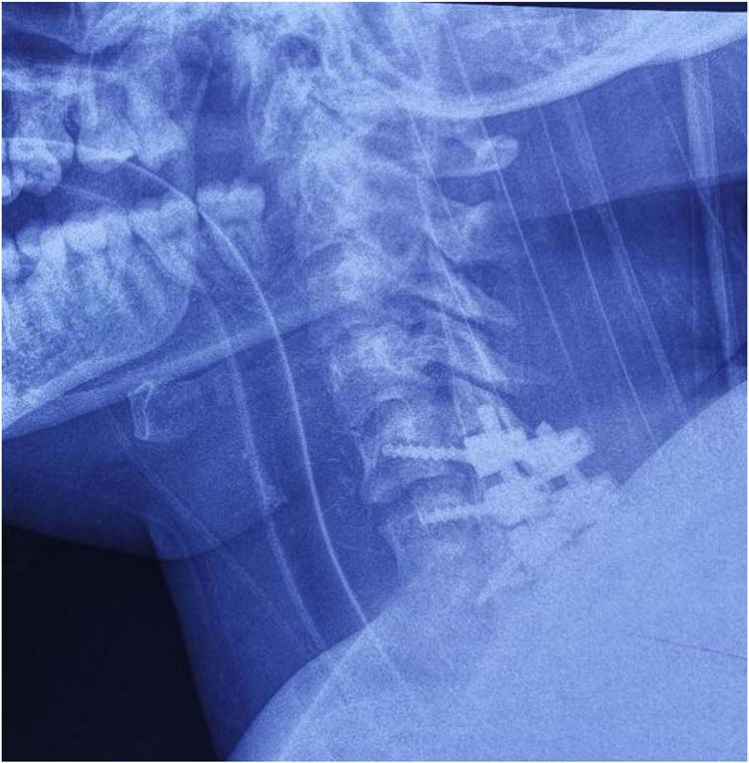
Cervical spine X-ray postoperatively – lateral view: properly positioned osteosynthesis material.

On the fourth day after the injury, the patient was conscious, on low doses of vasopressor support. What was surprising was that in her conscious state she had weakened abduction of the left shoulder, while the right arm and legs were completely plegic (ASIA A). We decided to perform posterior reduction and stabilization from the level of C5 to T1 (Figs 3–5). On the first postoperative day, a follow-up X-ray of the cervical spine was performed (Figs 6 and 7).

Several days after the surgery her body temperature increasing in the subsequent days to 39.9°C, there was an increase in markers of sepsis (PCT) and inflammatory markers (CRP, WBC) in the blood. On the 16th day after the injury, the patient’s overall condition deteriorated further, asystole was registered on the monitor, cardiac rhythm was not restored, despite the application of necessary measures of cardiopulmonary resuscitation, and the patient was pronounced deceased.

## Discussion

Transection of the spinal cord in the cervical region of the spine caused by traumatic dislocation is an injury that often leads to death, especially when it is a spinal cord injury in the upper cervical spine because of respiratory impairment and vascular lesions. Gupta showed the flexion/distraction injury is associated with vertebral artery injury [7], and on the other hand Biffl did not find a significant pattern in the mechanism of injury or the level of cervical spine injury associated with injury to the vertebral artery [8]. Spinal cord injuries caused by luxation and/or dislocation of the cervical spine, at the level C3-C7 vertebra, lead to motor and sensory paralysis below the level of the injury and sphincter dysfunction [9]. In the case of our patient, although the dislocation of the cervical spine was extreme, there was no injury to the major blood vessels (vertebral artery, common carotid artery) nor was there any injury to the surrounding vital structures (trachea, esophagus).

If an injured person survives a cervical spinal cord injury, it is vitally important to diagnose the injury immediately and perform early surgical treatment [10]. The ATLS protocol (Advanced Trauma Life Support protocol) is to be followed in the initial clinical evaluation of trauma patients suspected of having cervical spine and spinal cord injury [11].

In terms of early diagnosis of cervical spine injuries, there are numerous studies that have confirmed the much higher sensitivity and specificity of CT scanners as compared with conventional radiography, especially when there is injury at the craniocervical junction [12].

In the case of our patient, we had a dilemma as to when and how to perform the surgical procedure. Due to the poor overall condition of the patient, the surgical procedure was not performed within the first 24 hours upon injury, rather, medicamentous treatment of acute spinal cord injury with high doses of methylprednisolone was started, in keeping with the protocol [13]. After the patient was hemodynamically stabilized to an acceptable degree, and after injuries to vital structures had been excluded, we decided to perform the surgical procedure. Regarding time of surgery, Fehlings has recently found that decompression prior to 24 hours after SCI can be performed safely and is associated with improved neurologic outcome [14].

Dislocation reduction, spinal cord decompression, and stabilization of a dislocated segment in the neck can be achieved by the anterior and by the posterior surgical approach, or by combining these two approaches. We decided on the posterior approach, because we were of the opinion that it would be easier to achieve repositioning with this approach. Additionally, the patient had a marked hematoma on the front of the neck, which could have compromised the approach itself as well as postoperative healing of the wound. We had planned to perform additional anterior stabilization, based on postoperative recovery, but unfortunately, she died on the 16th day upon injury.

## Conclusion

To the best of our knowledge, such an injury has not been described in literature so far. The injury itself threatened the patient’s life from the very beginning. In such rare extreme dislocations, the decision must be made on a case-by-case basis, and founded on protocols, teamwork and, ultimately, the experience of the surgeon performing the procedure.

## References

[1] Petrone B , Dowling TJ. Cervical dislocation. [Updated 2023 May 22]. In: StatPearls [Internet]. Treasure Island (FL): StatPearls Publishing, 2023 Jan. Available from: https://www.ncbi.nlm.nih.gov/books/NBK557528/32491460

[2] Astur N , Klimo P Jr, Sawyer JR, et al. Traumatic atlanto-occipital dislocation in children: evaluation, treatment, and outcomes. J Bone Joint Surg Am 2013;95:e194. 10.2106/JBJS.L.01295. 24352780

[3] Sribnick EA , Hoh DJ, Dhall SS. Traumatic high-grade cervical dislocation: treatment strategies and outcomes. World Neurosurg 2014;82:1374–9. 10.1016/j.wneu.2014.02.008. 24530458

[4] Mubark I , Abouelela A, Hassan M, et al. Sub-axial cervical facet dislocation: a review of current concepts. Cureus 2021;13:e12581. 10.7759/cureus.12581. 33575145 PMC7870112

[5] Cao BH , Wu ZM, Liang JW. Risk factors for poor prognosis of cervical spinal cord injury with subaxial cervical spine fracture-dislocation after surgical treatment: a CONSORT study. Med Sci Monit 2019;25:1970–5. 10.12659/MSM.915700. 30877267 PMC6433098

[6] Quarrington RD , Jones CF, Tcherveniakov P, et al. Traumatic subaxial cervical facet subluxation and dislocation: epidemiology, radiographic analyses, and risk factors for spinal cord injury. Spine J 2018;18:387–98. 10.1016/j.spinee.2017.07.175. 28739474

[7] Gupta R , Siroya HL, Bhat DI, et al. Vertebral artery dissection in acute cervical spine trauma. J Craniovertebr Junction Spine 2022;13:27–37. 10.4103/jcvjs.jcvjs_3_22. 35386245 PMC8978858

[8] Biffl WL , Moore EE, Elliott JP, et al. The devastating potential of blunt vertebral arterial injuries. Ann Surg 2000;231:672–81. 10.1097/00000658-200005000-00007. 10767788 PMC1421054

[9] Hadley MN , Walters BC, Aarabi B, et al. Clinical assessment following acute cervical spinal cord injury. Neurosurgery 2013;72:40–53. 10.1227/NEU.0b013e318276edda. 23417178

[10] Okereke I , Mmerem K, Balasubramanian D. The management of cervical spine injuries – a literature review. Orthop Res Rev 2021;13:151–62. 10.2147/ORR.S324622. 34611449 PMC8487293

[11] Schmidt OI , Gahr RH, Gosse A, Heyde CE. ATLS and damage control in spine trauma. World J Emerg Surg 2009;4:9. 10.1186/1749-7922-4-9. 19257904 PMC2660300

[12] Hoffman JR , Mower WR, Wolfson AB, et al. Validity of a set of clinical criteria to rule out injury to the cervical spine in patients with blunt trauma. National Emergency X-Radiography Utilization Study Group. N Engl J Med 2000;343:94–9. 10.1056/NEJM200007133430203. 10891516

[13] Zagorac S . Methylprednisolone therapy in acute spinal cord injuries. Serbian J Med Chamb 2021;2:409–15. 10.5937/smclk2-34472.

[14] Fehlings MG , Vaccaro A, Wilson JR, et al. Early versus delayed decompression for traumatic cervical spinal cord injury: results of the Surgical Timing in Acute Spinal Cord Injury Study (STASCIS). PloS One 2012;7:e32037. 10.1371/journal.pone.0032037. 22384132 PMC3285644

